# *Borassus aethiopum*-Fortified Bread Reduces Metabolic Risk Factors among Cardiovascular Disease Outpatients at 37 Military Hospital, Accra: A Pilot Study

**DOI:** 10.1155/2020/2379061

**Published:** 2020-07-19

**Authors:** Charles Apprey, Marian Peprah, Reginald Adjetey Annan, Marina A. Tandoh, Odeafo Asamoah-Boakye

**Affiliations:** Department of Biochemistry and Biotechnology, Faculty of Biosciences, College of Science, Kwame Nkrumah University of Science and Technology, PMB KNUST, Kumasi, Ghana

## Abstract

**Background:**

Dyslipidemia and hypertension are the leading causes of morbidity and mortality in patients with cardiovascular diseases (CVDs).

**Objective:**

The study sought to evaluate the effects of *Borassus aethiopum*-fortified bread on metabolic risk factors among CVD outpatients.

**Method:**

From August 2016 to April 2017, a pilot study using a single-blinded randomized placebo-controlled trial was conducted by administering *Borassus*-fortified bread (150 g) and indistinguishable placebo (150 g white flour bread) daily to 122 CVD outpatients at 37 Military Hospital, Accra, Ghana, for 90 days. Body composition, blood pressure, and biochemical parameters were evaluated before and after the intervention.

**Results:**

Following the intervention, the mean waist circumference (before: 98.3 ± 14.6 cm, after: 95.9 ± 15.8 cm, *P* = 0.030), BMI (before: 31.4 ± 6.9 kg/m^2^, after: 28.0 ± 5.8 kg/m^2^, *P* = 0.027), and visceral fat (before: 10.4 ± 3.2, after: 9.9 ± 3.0, *P* = 0.013), as well as systolic (from 161.2 ± 25.5 to 137.6 ± 22.9and diastolic (from 99.2 ± 13.6 to 85.1 ± 10.8) blood pressure, were significantly reduced among the experimental group. Likewise, serum total cholesterol (TC), LDL, and HDL were significantly reduced within the experimental group before (TC: 5.9 ± 1.1, LDL: 3.4 ± 1.1, and HDL: 2.2 ± 0.5) and after the intervention (TC: 4.9 ± 1.1, LDL: 2.8 ± 0.9, and HDL: 1.5 ± 0.4) (TC: *P* = 0.001, LDL: *P* = 0.016, and HDL: *P* < 0.001, in mmol/L). These reductions were not observed in the controls.

**Conclusion:**

The *Borassus*-fortified bread significantly reduced blood pressure and improved lipid profile and other metabolic risk factors among the CVD outpatients studied. Therefore, its potential in the management of CVDs and other metabolic-related diseases should be looked at.

## 1. Introduction

Functional foods from plants (quinoa, inulin) can be consumed with diet to provide added nutritional benefits including cholesterol-lowering and hypoglycemic effects, which can be used to manage health conditions such as cardiovascular disease and diabetes [1, 2]. *Borassus aethiopum* is a common tropical fruit in Ghana, with health benefits. Studies have shown that *Borassus* fruit pulp contains phytochemicals: flavonoids, alkaloids, triterpenes, steroids and sterols (cardiac glycosides), saponins, and phenols [3–7], as well as substantial antioxidant levels [8, 9]. Also, a study done by Issaka et al. [4] exploited the antidiabetic effect of the *Borassus* fruit in a rat model, and the findings provided enough evidence to test for the hypothesis that *Borassus*-fortified bread could provide health benefits on the metabolic risk parameters among patients with CVDs. In our previous study, we found that non-nutritive components including phytochemicals were still present in *Borassus*-fortified bread and could provide potential health benefits when consumed [8]. In Ghana, the fruit is seasonally available, abundant, and inexpensive [10]; however, its rich phytochemicals and antioxidant properties have not been extensively exploited to show its benefits among people with cardiovascular diseases.

Cardiovascular diseases (CVDs) remain the leading non-communicable diseases, causing morbidity and mortality across the globe [11]. CVDs result from malfunctioning of the heart and blood vessels at both micro- and macrolevels [11, 12]. They include hypertension, coronary heart disease, cerebrovascular accidents (stroke), and rheumatic heart disease [14], and it is prevalent among the Ghanaian population [15].

Globally, the World Health Organization estimated that 17.5 million people died from the disease in 2015 [13]. Of these deaths, an estimated 7.4 million and 6.7 million were due to coronary artery disease (CAD) and stroke, respectively [13]. In sub-Saharan African countries, the burden of cardiovascular diseases has seen a steady increase, with CVD-related deaths accounting for almost 9.2% of all deaths [16]. In Ghana, the situation is the same and coronary heart disease accounts for 6.5% death annually [12]. In Ghana, CVDs have increasingly become the top most public health concern, which needs proactive measures to address the menace [17].

Most studies have reported that increasing age, high body mass index, physical inactivity, unhealthy diet, high blood pressure (hypertension), dyslipidemia, diabetes mellitus, overweight or obesity, oxidative stress, and family history are associated with the occurrence of cardiovascular diseases [18–20]. Also, a study by Nkum and Micah [15] revealed that obesity, physical inactivity, smoking, and reduced consumption of fruits and vegetables were common risk factors for developing cardiovascular diseases among the Ghanaian population. According to Addo et al. [17], being either a hypertensive or a prehypertensive diabetic patient, with concurrent dyslipidemia, highly increased the risk of developing cardiovascular disease in the Ghanaian population. Among people with diabetes, it is reported that having dyslipidemia, secondary to uncontrolled hyperglycemia, increases the risk of proatherogenic and subsequently may lead to the development of cardiovascular diseases [21]. Furthermore, Boateng et al. [22] estimated a 10-year risk of CVDs using the pooled cohort equation with estimates of ≥7.5% defining high CVD risk in the Ghanaian rural and urban populations. The study found that, among the Ghanaian men, the proportion with CVD risk ≥ 7.5% was 34.7% in rural Ghana and 45.4% in urban Ghana. The contributing risk factors for CVDs in the study were age, sex, systolic blood pressure, high total cholesterol, high-density lipoprotein, and diabetes mellitus diagnosis [22]. Even though cross-sectional studies in Ghana had revealed some CVD risk factors [15, 17], no data exist on intervention studies, targeting how to reduce the risk factors associated with CVDs.

The pathogenesis of atherosclerosis in the initiation and progression of CVDs is complicated and has been linked to influences of obesity and abdominal obesity on blood lipids [23, 24]. Alterations in lipids and lipoprotein parameters contribute to oxidative stress and development of atherosclerosis [25]. The end product of this oxidative stress is reactive oxygen species (ROS) identified to oxidize LDL to cause endothelial damage [5]. The dysfunction of endothelial tissue can stimulate atherosclerotic events in blood vessels which can progress to cause cardiovascular diseases [14]. The literature suggests that natural plants (e.g., purslane, cladodes of prickly pear) possess great antioxidant, cholesterol-reducing, and hypoglycemic properties, mainly due to their phenolic content which can promote cardiovascular functions to humans and thus can be used as food [26, 27]. However, the rich phytochemicals and antioxidant properties of the *Borassus* fruit could potentially reduce cardiovascular disease risk factors among people with CVDs [6]. Hence, this study is aimed at evaluating the effects of *Borassus aethiopum*-fortified bread on metabolic risk factors among cardiovascular disease outpatients at 37 Military Hospital, Accra.

## 2. Materials and Methods

### 2.1. Study Design

The study was conducted at the medical department of 37-Military Teaching Hospital, which is the second largest medical facility in Accra, the capital city of Ghana. It is a 600-bed capacity hospital that manages about 1000 newly diagnosed and old cardiovascular patients across Ghana annually. The medical department has an outpatient unit that provides 24-hour medical services to the general population.

### 2.2. Study Population

The study included only outpatients diagnosed with cardiovascular diseases. The participants were known CVD outpatients who were at various stages of CVDs such as dyslipidemia, hypertension, stroke, and diabetes (impaired glucose tolerance). A total of about 1000 patients report at the study site annually including newly diagnosed and old cases.

### 2.3. Eligibility

All participants aged 18 years and above, diagnosed with cardiovascular disease, attending and receiving medical treatment at the medical department of 37-Military Teaching Hospital, were included in the study. CVD outpatients on medication at time of data collection were included. Participants from other departments and diagnosed with diseases other than CVDs and inpatients with CVDs were excluded from this study. Participants who met inclusion criteria but did not provide written informed consent, pregnant women, and mentally unstable patients were also excluded from this study.

### 2.4. Sample Size

Cochran's formula (1989) was used, *N* = *Z*^2^ *p* (1 − *p*)/*d*^2^, where *N* represents the sample size, *Z* is the confidence level (95%) (*Z*-score standard value = 1.96), *p* is the estimated cardiovascular disease (patient) prevalence (0% = 0.1), *d* is the marginal error, and *N* = 138.

A total of 138 participants should have been used; however, in anticipation of dropouts, 150 participants were recruited for the study. Finally, the sample size was reduced to 122, due to noncompliance, dropouts, missing contact, and death. The sample size was calculated based on primary outcome data on CVDs.

### 2.5. Ethics

The 37 Military Hospital Institutional Review Board and Committee on Human Research, Publication and Ethics approved the protocols and gave a clinical registry number (37MH-IRB IPN 082/2016) before commencement of the study. Also, approval from the Committee on Human Research, Publication and Ethics at the School of Medical Sciences and Komfo Anokye Teaching Hospital was obtained with the ethical number, CHRPE/AP/450/16. The purpose of the study was made known to all participants. Assurance was also given to the participants of their confidentiality of information given, and finally, their consent was sought by the signing of consent forms. There was no discrimination whatsoever among participants, and any information got was not identifiable.

### 2.6. Data Collection and Sampling Method

Participants were recruited consecutively using simple random sampling. Cardiovascular disease patients who attended the hospital between September and November 2016 were randomly selected after medical assessment by a cardiology doctor. It took sixty days to recruit participants to be enrolled in the study. After enrolment, the study used 90 days from baseline assessment to end of intervention. Baseline data was collected in December 2016, and intervention continued from December 2016 to February 2017.

#### 2.6.1. Randomization

A randomized single-blinded placebo-controlled clinical trial method was employed for the study. The participants were assigned to each group (experimental and control) using simple randomization in a 1 : 1 ratio. An unknown person was invited and blindfolded to write codes (representing the experimental and control groups) to be given to participants. Participants were placed in a draw, with two people pulled from the draw alternatively, with each person representing a group (experimental or control).

#### 2.6.2. Intervention

**Figure 1:**
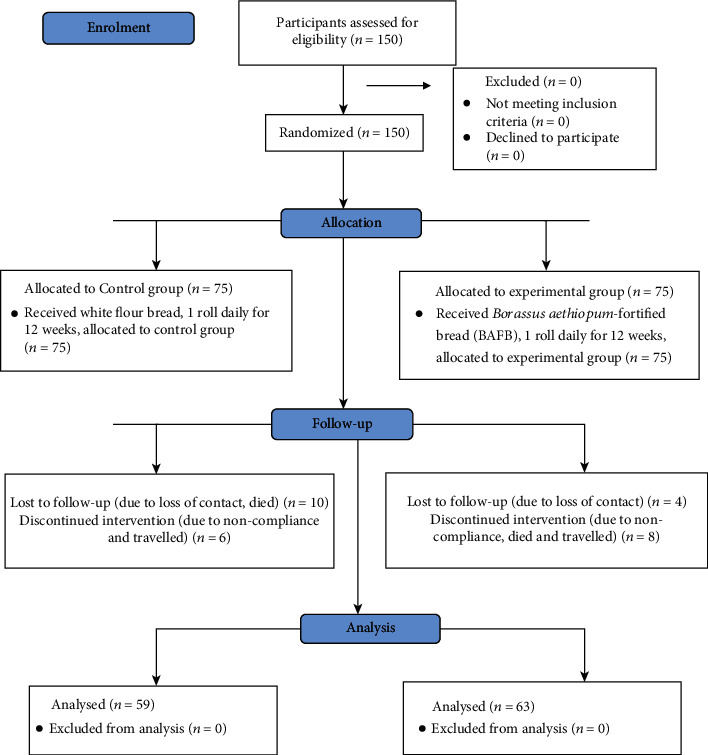
Flow chart of the study design and procedure.

White flour was used in a varied ratio with the RBAP. The ratio of white flour to RBAP was 2 : 1 (66.7% : 33.3%), 5 : 1 (83.3% : 16.7%), 10 : 1 (90.9% : 9.1%), and 20 : 1 (95.2% : 4.8%) giving a total of 4 samples. The type of bread was locally made Ghanaian tea bread (made from white flour) and tea bread enriched with raw *Borassus aethiopum* powder. Each participant of the experimental group received 150 g of *Borassus*-fortified bread daily for three months. 150 g of white flour bread as a placebo was also given to the control group daily for three months, and this bread (150 g white flour bread) was prepared with the same ingredients as used for the intervention except for raw *Borassus aethiopum* powder (RBAP). Also, 5 grams of raw *Borassus aethiopum* powder was added to the *Borassus*-fortified bread for daily consumption by the experimental group. The size, shape, weight, and packaging of both the *Borassus*-fortified bread and the white flour bread were similar. The *Borassus*-fortified bread was given to the experimental group, to evaluate the impact of the bread in lowering blood lipid and other metabolic parameters. Participants in both the control and experimental groups reported no change to their usual dietary pattern during the study period except the inclusion of white flour bread or *Borassus*-fortified bread. The amount of bread administered, the timings involved, and the quantity of *Borassus aethiopum* flour in the bread were monitored and supervised regularly by the researcher. Participants were not aware of what was in the bread given at any time. Constituents of both types of bread were only known by the researcher. Details of the experimental design are presented in Figure 1. Participants also received intensive clinical monitoring through consultation with their doctor monthly, for three months. In addition to that, some participants (*N* = 46) had some changes made to their drug therapy by their physicians during the study period. These included patients in both the experimental (*N* = 28) and control (*N* = 18) groups.

#### 2.6.3. Outcome Measures

The primary outcome measures were anthropometric measurements (duplicate), which were done at baseline and three months (end of the study) by the same person who was blinded to the groups. Secondary outcome measures included blood pressure and lipid profile. At baseline, a questionnaire was administered to collect demographic data, medical history, drugs (atorvastatin: 10 mg, 20 mg; Crestor: 10 mg, 20 mg; and simvastatin: 40 mg), and social history. Also, 28 participants were on various statin therapies and were unknowingly placed among the experimental and control groups. Anthropometric measurement included height (using a stadiometer, Seca 213, Germany), weight, body mass index (BMI), visceral fat, metabolic age (using Omron BF511, India), and waist circumference (using a tape measure). Blood pressure (quadruplets) was obtained at baseline and every month using a manual sphygmomanometer and stethoscope and was done under standardized conditions according to WHO guidelines [28]. Anthropometric data and biochemical data (lipid profile) were also done at two-time points: baseline and end of intervention.

At baseline and three months, dietary intakes using 24-hour dietary recall were collected from participants but this is not reported in the results for this study. Also, there were no changes to their dietary intakes throughout the study from the 24-hour dietary recall reported by participants, except the inclusion of *Borassus*-fortified bread and white flour bread.

At baseline and three months, 5 mL venous blood samples were taken and placed into gel activator tubes. The blood samples stood for about 10 minutes and centrifuged at 4000 rpm for 5 minutes. 2 mL of blood serum was pipetted into cyrotubes and placed in a cold compartment below 0°C. A fully automated ACE Alera Clinical Chemistry Analyzer and its reagent, manufactured by Alfa Wassermann Inc., were used to analyze high-density lipoprotein (HDL), total cholesterol (TC), triglyceride (TG), and low-density lipoprotein (LDL).

#### 2.6.4. Quality Control

Research assistants were trained on anthropometric instruments to ensure that they have maximum control over the research data collection. All the data collection tools and equipment were pretested and calibrated, respectively, before data collection. This was done to assess the feasibility and acceptability of the study procedures.

### 2.7. Data Analysis

Data was analyzed on Statistical Package for the Social Sciences (SPSS version 23, Chicago, IL). A chi-squared, crosstabulation test was performed to compare sociodemographic variables (gender, age, financial status, family history, social history, and diagnoses) and study groups (experimental and control), and values were reported as frequency and percentage and chi-squared *P* value (Fisher's exact *P* value). A comparison of anthropometric, biochemical data of the intervention and control groups was done using an independent sample *t*-test and paired *t*-test, and values were reported as mean ± standard deviation for continuous parameters (BMI, waist circumference, visceral fat, blood pressure, and lipid profile). A test on normality showed the Shapiro-Wilk *P* value greater than 0.05 for all variables, which means variables were normally distributed (Table 1). All analyses were 2-tailed, and *P* values < 0.05 were considered statistically significant.

## 3. Results

### 3.1. Sociodemographic Characteristics of Participants

A total of 122 participants were recruited for this study, 63.9% were female and 36.1% male. The majority (59.8%) of the participants were 50 years and above. Medically, all the participants were diagnosed with cardiovascular diseases but presented comorbidities, of whom 51.6% of them had hypertension alone with 1.6% of them diagnosed with a stroke. Also, 5% of participants were past tobacco users and less than 1 percent (0.8%) took alcoholic beverages at the time of data collection (Table 2).

### 3.2. Effect of *Borassus aethiopum*-Fortified Bread on Anthropometric Parameters of Participants

There was a significant difference in the mean waist circumference (before: 98.3 ± 14.6 cm versus after: 95.9 ± 15.8 cm, *P* = 0.030), BMI (before: 31.4 ± 6.9 kg/m^2^ versus after: 28.0 ± 5.8 kg/m^2^, *P* = 0.027), visceral fat (before: 10.4 ± 3.2 versus after: 9.9 ± 3.0,*P* = 0.013), and metabolic age (before: 61.6 ± 13.4 years versus after: 59.3 ± 13.0 years, *P* = 0.010) among the experimental group However, no significant difference in BMI, WC, VF, and metabolic age was found between the control and experimental groups before and after intervention (*P* > 0.05) (Table 3).

### 3.3. Effect of *Borassus aethiopum*-Fortified Bread on Blood Pressure of Participants

The mean systolic and diastolic blood pressure values measured at four different points in the three-month period are presented in Table 4. The mean systolic blood pressure was reduced significantly in the experimental group than the control group in the third (*P* = 0.005) and fourth readings (*P* = 0.005). Compared with the control group, the experimental group had significantly lower mean systolic blood pressure in the third (*P* = 0.002) and fourth monthly readings (*P* = 0.009). Also, there was a significant reduction in systolic and diastolic blood pressure before (systolic: 161.2 ± 25.5 mmHg; diastolic: 99.2 ± 13.6 mmHg) and after (systolic: 137.6 ± 22.9 mmHg; diastolic: 85.1 ± 10.8 mmHg) the intervention in the experimental group (systolic: *P* = 0.005; diastolic: *P* = 0.009) (Table 4).

### 3.4. Effect of *Borassus aethiopum*-Fortified Bread on the Lipid Profile of Participants

Table 5 presents the effects of the *Borassus*-fortified bread on the lipid profile of participants by groups. Before and after the intervention, there was no significant difference in total cholesterol (TC), triglyceride (TG), high-density lipoprotein (HDL), and low-density lipoprotein (LDL) in the control groups. There was a significant reduction in TC, LDL, and HDL before (TC: 5.9 ± 1.1 mmol/L, LDL: 3.4 ± 1.1 mmol/L, and HDL: 2.2 ± 0.5 mmol/L) and after the intervention (TC: 4.9 ± 1.1 mmol/L, LDL: 2.8 ± 0.9 mmol/L, and HDL: 1.5 ± 0.4 mmol/L) in the experimental group (TC: *P* = 0.001, LDL: *P* = 0.016, and HDL: *P* < 0.001). Also, after the intervention, there was a significant difference in TC, LDL, and HDL between the experimental and control groups (TC: *P* = 0.001, LDL: *P* = 0.015, and HDL: *P* < 0.001) (Table 5).

Table 1 presents the nutrient components of the white flour bread and *Borassus*-fortified bread. The nutrient composition for the white flour bread and *Borassus*-fortified bread was similar except that *Borassus*-fortified bread contained more dietary fiber and other phytonutrients (phytochemicals) including flavonoids, saponins, glycosides, triterpenes, phenolics, steroids and sterols, and vitamin C (Table 1).

## 4. Discussion

In the present study, we evaluated the effects of *Borassus*-fortified bread on metabolic risk factors of Ghanaian cardiovascular disease outpatients. The *Borassus*-fortified bread significantly reduced levels of systolic and diastolic blood pressure, TC, and LDL of CVD outpatients during the 90-day supplementation. Also, the consumption of *Borassus*-fortified bread helped improve waist circumference, visceral fat, metabolic age, and BMI, reflecting overall health benefits for the CVD patients. Both groups had no diet restriction over 90-days, but the experimental group consumed 150 g *Borassus*-fortified bread. Findings revealed that females were dominant than males in this study. CVD is known to affect older adults [29], and the finding revealed that close to 6 out of 10 participants (58.9%) were 50 years and above. The CVD participants also presented with comorbidities associated with the disease, such as hypertension (51.6%), hypertension and high blood cholesterol (26.2%), and stroke (0.8%).

It is well established that obesity (central adiposity) is associated with chronic diseases such as cardiovascular diseases, diabetes, and hypertension [30]. In the present study, there was a significant difference in the mean waist circumference (*P* = 0.030), BMI (*P* = 0.027), visceral fat (*P* = 0.013), and metabolic age (*P* = 0.010) among the experimental group. The reduction in waist circumference, visceral fat, and BMI can be explained by the higher fiber content of the *Borassus*-fortified bread, as also found in a study by Sudhakara et al. [31], and this might have influenced the reduction in fat absorption. The overall reduction of WC, VF, and BMI, together with the presence of antioxidants in the raw *Borassus aethiopum* powder, might have influenced the decrease in metabolic age among the experimental group.

Findings revealed a significant reduction in systolic blood pressure in the experimental group than the control group in the third (*P* = 0.005) and fourth monthly readings (*P* = 0.005). Also, compared with the control group, the experimental group had significantly lower mean systolic blood pressure in the third (*P* = 0.002) and fourth monthly readings (*P* = 0.009). The reduction in systolic blood pressure can be attributed to the presence of phytochemicals such as flavonoids in the raw *Borassus aethiopum* powder as shown in the study by Peprah et al. [8]. Flavonoids are known to decrease the formation of atherosclerotic plaques and reduce arterial stiffness, making arteries sensitive to stimuli of vasodilation and thus ensuring blood flow under normal pressure [32].

High low-density lipoprotein and hypercholesterolemia are known major risk factors for developing cardiovascular diseases, while high-density lipoprotein is protective of cardiovascular diseases [33]. The findings revealed no significant change in the lipid profile in the control group throughout the study, while total cholesterol, low-density lipoprotein, and high-density lipoprotein were significantly reduced in the experimental group (TC: *P* = 0.001, LDL: *P* = 0.016, and HDL: *P* < 0.001). Also, after the intervention, there was a significant difference in TC, LDL, and HDL between the experimental and control groups (TC: *P* = 0.001, LDL: *P* = 0.015, and HDL: *P* < 0.001). This contradicts the findings by Issaka et al. [4], who reported no significant change in the lipid profile levels of both the normal and diabetic rats, in addition to no significant changes in their coronary risk factors. This could be attributed to the shorter duration of their study (28 days) compared to our study (90 days). Furthermore, some studies have shown the presence of phytochemicals such as saponins, tannins, and terpenoids in the aqueous extract of *Borassus* [3], while flavonoids, sterols, and saponins were also found in *Borassus*-fortified bread ^6^, and these could have potentially influenced the decrease in systolic and diastolic blood pressure, total cholesterol, and low-density lipoprotein in the experimental group. Saponins possess membranolytic property which inhibits absorption of cholesterol and facilitates its excretion through the intestinal tract [8]. The *Borassus*-fortified bread also contains a substantial quantity of antioxidants [8], which could exhibit a cardiovascular protective role in the experimental group, leading to the reduced circulatory TC and LDL levels. However, the *Borassus*-fortified bread had lesser effect on the HDL level of participants.

To the best of our knowledge, this study is the first experimental human study to establish the effects of *Borassus*-fortified bread on reducing metabolic risk parameters of cardiovascular disease outpatients. Hence, further similar intervention studies are needed to confirm the findings of the present study. This study has proven to some extent that consumption of *Borassus*-fortified bread can significantly reduce metabolic risk factors associated with CVDs in cardiovascular disease patients. The *Borassus* fruit is inexpensive and is abundant in Ghana, and it has proven to have cardioprotective benefits when used as a fortificant in bread. The *Borassus*-fortified bread is inexpensive, and the fruit is abundant in Ghana and has been proven to have cardioprotective benefits.

## 5. Conclusion

The *Borassus*-fortified bread significantly reduced levels of systolic and diastolic blood pressure, TC, and LDL of CVD outpatients during the 90-day supplementation. The consumption of *Borassus*-fortified bread also helped improve waist circumference, visceral fat, metabolic age, and BMI, reflecting overall health benefits for the CVD patients. The consumption of *Borassus*-fortified bread could potentially help in the management of CVDs. There is therefore a need for industrial utilization of the *Borassus* fruit to fortify other products as it could provide health benefits in the management of people with cardiovascular diseases as well as serve as a potential protective food for chronic diseases.

## Figures and Tables

**Table 1:** Test of normality of variables.

Variables	Control group	Shapiro-Wilk *P* value	Experimental group	Shapiro-Wilk *P* value
WC		0.855		0.065
BMI		0.133		0.060
VF		0.485		0.214
Metabolic age		0.080		0.071
TC		0.057		0.068
TG		0.240		0.130
HDL		0.595		0.666
LDL		0.310		0.342

**Table 2:** Baseline characteristics of participants by group.

Parameters	Total (*N* = 122)	Group		*P* value
		Control (*N* = 59)	Experimental (*N* = 63)	
Age (male)				**<0**.**001**
19-29	2 (1.6)	1 (45)	1 (45)	
30-39	8 (6.6)	6 (27.3)	2 (9.1)	
40-49	7 (5.7)	4 (18.2)	3 (13.6)	
50+	27 (22.1)	11 (50)	16 (72.7)	
Age (female)				0.100
19-29	2 (1.6)	0 (0.0)	2 (4.9)	
30-39	7 (5.7)	4 (10.8)	3 (7.3)	
40-49	23 (18.9)	15 (40.5)	8 (19.5)	
50+	46 (37.7)	18 (48.6)	28 (68.3)	
Gender				0.467
Female	78 (63.9)	37 (47.4)	41 (52.5)	
Male	44 (36.1)	22 (50.0)	22 (50.0)	
Comorbidities				0.122
Chol	6 (4.9)	3 (50.0)	3 (50.0)	
Chol, DM	1 (0.8)	1 (100.0)	0 (0.0)	
Chol, Hpt	32 (26.2)	11 (33.3)	21 (66.6)	
Chol, Hpt, DM	8 (6.6)	2 (25.0)	6 (75.0)	
Hpt	63 (51.6)	37 (58.7)	26 (41.2)	
Hpt, DM	9 (7.4)	5 (55.5)	4 (44.4)	
Hpt, stroke	1 (0.8)	0 (0.0)	1 (100.0)	
Stroke	1 (0.8)	0 (0.0)	1 (100.0)	
Social history				
Tobacco use				0.628
Tobacco users	116 (9)	56 (48.3)	60 (51.7)	
Nontobacco users	6 (5)	3 (50.0)	3 (50.0)	
Alcohol intake				0.484
Drink alcohol	121 (99.2)	58 (47.9)	63 (52.0)	
Nonalcoholic	1 (0.8)	1 (100.0)	0 (0.0)	
Medication	46 (37.7)	*N* = 18	*N* = 28	0.890
Atorvastatin (10 mg)	8 (28.6)	7 (38.9)	15 (32.6)	
Atorvastatin (20 mg)	7 (25.0)	4 (22.2)	11 (23.9)	
Crestor (10 mg)	6 (21.4)	6 (33.3)	12 (26.1)	
Crestor (20 mg)	5 (17.9)	1 (5.6)	6 (13.0)	
Simvastatin (40 mg)	2 (7.1)	0 (0.0)	2 (4.3)	

Data shows frequencies (percentages) of categorical variables; some cells were less than 5 so Fisher's exact *P* value was reported. Chol: cholesterol; DM: diabetes mellitus; Hpt: hypertension. Others: pastor, hairdresser, and artisan. The bold value is significant at *P* < 0.05.

**Table 3:** Comparison of anthropometric characteristics of participants by groups.

Anthropometric parameter	Group					
	Control (*N* = 59)		*P* value	Experimental (*N* = 63)		*P* value
	Before	After		Before	After	
WC (cm)	96.0 ± 15.9	95.1 ± 15.7	0.425	98.3 ± 14.6	95.9 ± 15.8	**0**.**030**
BMI (kg/m^2^)	29.9 ± 7.2	28.0 ± 6.1	0.212	31.4 ± 6.9	28.0 ± 5.8	**0**.**027**
Visceral fat	9.8 ± 4.2	10.0 ± 3.9	0.325	10.4 ± 3.2	9.9 ± 3.0	**0**.**013**
Metabolic age (years)	59.1 ± 16.6	58.9 ± 15.8	0.820	61.6 ± 13.4	59.3 ± 13.0	**0**.**010**

Anthropometric parameter	Before intervention		*P* value	After intervention		*P* value
	Control	Experimental		Control	Experimental	
WC (cm)	96.0 ± 15.9	98.3 ± 14.6	0.412	95.1 ± 15.7	95.8 ± 15.8	0.785
BMI (kg/m^2^)	29.9 ± 7.2	31.4 ± 6.9	0.275	28.0 ± 6.1	28.0 ± 5.8	0.920
Visceral fat	9.8 ± 4.2	10.4 ± 3.2	0.405	10.0 ± 3.9	9.9 ± 3.0	0.896
Metabolic age (years)	59.1 ± 16.0	61.6 ± 13.4	0.375	58.9 ± 15.8	59.3 ± 13.3	0.883

Paired *t*-test was performed. Data are presented as mean ± SD (standard deviation). Bold values are significant at *P* < 0.05.

**Table 4:** Comparison of blood pressure readings over four months of participants by study groups.

Blood pressure (mmHg)	Readings (monthly)		Group			*P* value
			Control (*N* = 59)	Experimental (*N* = 63)		
Systolic	1 (baseline)		156.7 ± 21.4	161.2 ± 25.5		0.298^¥^
	2		150.0 ± 21.4	145.1 ± 22.0		0.218^¥^
	3		152.4 ± 20.5	141.0 ± 23.5		**0**.**005**^¥^
	4		149.1 ± 21.1	137.6 ± 22.9		**0**.**005**^¥^
Diastolic	1 (baseline)		97.0 ± 12.5	99.2 ± 13.6		0.351^¥^
	2		92.9 ± 10.7	89.4 ± 13.2		0.119^¥^
	3		94.0 ± 9.4	87.8 ± 11.7		**0**.**002**^¥^
	4		89.7 ± 8.2	85.1 ± 10.8		**0**.**009**^¥^

Blood pressure (mmHg)	Group					
	Control (*N* = 59)		*P* value	Experimental (*N* = 63)		*P* value
	Before	After		Before	After	
Systolic	156.7 ± 21.4	149.1 ± 21.2	0.294	161.2 ± 25.5	137.6 ± 22.9	**0**.**005**^ǂ^
Diastolic	97.0 ± 12.5	88.7 ± 8.2	0.351	99.2 ± 13.6	85.1 ± 10.8	**0**.**009**^ǂ^

^¥^Independent *t*-test and ^ǂ^paired *t*-test were performed. Data are presented as mean ± SD (standard deviation). Bold values are significant at *P* < 0.05.

**Table 5:** Comparison of means of lipid profile of participants by groups.

Lipid profile (mmol/L)	Group					
	Control (*N* = 59)		*P* value	Experimental (*N* = 63)		*P* value
	Before intervention	After intervention		Before intervention	After intervention	
TC	5.8 ± 1.2	5.7 ± 1.2	0.567	5.9 ± 1.1	4.9 ± 1.1	**0**.**001**
LDL	3.4 ± 1.2	3.3 ± 1.2	0.893	3.4 ± 1.1	2.8 ± 0.9	**0**.**016**
TG	1.1 ± 0.4	1.1 ± 0.3	0.993	1.1 ± 0.4	1.3 ± 0.5	0.177
HDL	2.1 ± 0.6	1.9 ± 0.4	0.372	2.2 ± 0.5	1.5 ± 0.4	**<0**.**001**

Lipid profile (mmol/L)	Before intervention		*P* value	After intervention		*P* value
	Control	Experimental		Control	Experimental	
TC	5.9 ± 1.1	5.8 ± 1.2	0.569	5.7 ± 1.2	4.9 ± 1.1	**0**.**001**
LDL	3.4 ± 1.1	3.4 ± 1.3	0.893	3.4 ± 1.2	2.9 ± 0.9	**0**.**015**
TG	1.1 ± 0.4	1.1 ± 0.4	0.978	1.2 ± 0.3	1.3 ± 0.6	0.184
HDL	2.2 ± 0.5	2.1 ± 0.7	0.375	1.9 ± 0.5	1.5 ± 0.4	**<0**.**001**

Paired *t*-test was performed. Data are presented as mean ± SD (standard deviation). TC: total cholesterol; TG: triglyceride; HDL: high-density lipoprotein; LDL: low-density lipoprotein. Bold values are significant at *P* < 0.05.

**Table 6:** Nutrient composition of white flour bread and *Borassus*-fortified bread.

150 g of white flour bread (per 100 g)	150 g of *Borassus*-fortified bread (per 100 g)
Calories 265 kcal	Calories 265 kcal
Total fat 3.2 g	Total fat 3.2 g
Saturated fat 0.7 g	Saturated fat 0.7 g
Polyunsaturated fat 1.6 g	Polyunsaturated fat 1.6 g
Monounsaturated fat 0.6 g	Monounsaturated fat 0.6 g
Trans fat 0 g	Trans fat 0 g
Cholesterol 0 mg	Cholesterol 0 mg
Sodium 491 mg	Sodium 491 mg
Potassium 115 mg	Potassium 115 mg
Total carbohydrate 49 g	Total carbohydrate 49 g
Dietary fiber 2.7 g	Dietary fiber 5.4 g
Sugar 5 g	Sugar 5 g
Protein 9 g	Protein 9 g
Calcium 26%	Calcium 26%
Iron 19%	Iron 19%
Vitamin B_6_ 5%	Vitamin B_6_ 5%
Magnesium 6%	Magnesium 6%
	Phytochemicals present:
	Flavonoids^∗^
	Saponins^∗^
	Glycosides^∗^
	Triterpenes^∗^
	Phenolics^∗^
	Steroids and sterols^∗^
	Antioxidant present: vitamin C^∗^

^∗^Quantity not measured.

## Data Availability

The datasets used and/or analyzed during the current study are available from the corresponding author on reasonable request.

## Additional Points

*Limitations*. This study did not have total control on the dietary intakes and physical activity of the study participants during the 3-month study period which could be confounders to the study outcome. The differences in the effect of *Borassus*-fortified bread on the study participants based on their different drug treatments were also not assessed, but the control groups were on similar medications. *Miscellaneous Result.* In Table 6, the normality test shows that study data were normally distributed, since the Shapiro-Wilk *P* values were >0.05 in both the control and experimental groups.
